# Ivermectin to reduce malaria transmission III. Considerations regarding regulatory and policy pathways

**DOI:** 10.1186/s12936-017-1803-2

**Published:** 2017-04-24

**Authors:** Carlos Chaccour, N. Regina Rabinovich

**Affiliations:** 1 ISGlobal, Barcelona Ctr. Int. Health Res. (CRESIB), Hospital Clínic-Universitat de Barcelona, Barcelona, Spain; 2 Centro de Investigação Em Saúde de Manhiça, Maputo, Mozambique; 3 Instituto de Salud Tropical Universidad de Navarra, Pamplona, Spain; 4 Harvard T.H. Chan School of Public Health, Boston, MA USA

**Keywords:** Ivermectin, Endectocide, Regulatory pathway, Malaria, Policy, *Anopheles*

## Abstract

Vector control is a task previously relegated to products that (a) kill the mosquitoes directly at different stages (insecticides, larvicides, baited traps), or (b) avoid/reduce human-mosquito contact (bed nets, repellents, house screening), thereby reducing transmission. The potential community-based administration of the endectocide ivermectin with the intent to kill mosquitoes that bite humans, and thus reduce malaria transmission, offers a novel approach using a well-known drug, but additional steps are required to address technical, regulatory and policy gaps. The proposed community administration of this drug presents dual novel paradigms; first, indirect impact on the community rather than on individuals, and second, the use of a drug for vector control. In this paper, the main questions related to the regulatory and policy pathways for such an application are identified. Succinct answers are proposed for how the efficacy, safety, acceptability, cost-effectiveness and programmatic suitability could result in regulatory approval and ultimately policy recommendations on the use of ivermectin as a complementary vector control tool.

**Electronic supplementary material:**

The online version of this article (doi:10.1186/s12936-017-1803-2) contains supplementary material, which is available to authorized users.

## Background

One key goal of research on the use of ivermectin to reduce malaria transmission is documentation of the evidence required to support recommendation by the National Malaria Control Programmes (NMCP). Clearly, both safety and efficacy (defined as public health impact) would need to be established. There are, however, additional knowledge gaps that would need to be resolved to support regulatory approval and a World Health Organization (WHO) policy recommendation. This third paper of the thematic series reviews the main knowledge gaps in these key aspects.

## Regulatory pathways

### Current regulatory approval status

The current ivermectin oral formulation has FDA approval for the treatment of strongyloidiasis (200 mcg/kg single dose) and for the treatment/control of onchocerciasis in mass-distribution campaigns (150 mcg/kg one to four times a year) [1]. Ivermectin is additionally approved for the treatment of head lice [2] and rosacea [3] as topical formulations.

The French regulatory authorities have approved ivermectin for treatment of the microfilaraemia caused by *Wuchereria bancrofti* (150–200 mcg/kg twice a year or 300–400 mcg/kg once a year), strongyloidiasis (200 mcg/kg single dose) and scabies (200 mcg/kg once or twice in a 2-week period) [4]. In addition to onchocerciasis and strongyloidiasis, the Australian Therapeutic Drugs Administration has approved oral ivermectin for the treatment scabies (200 mcg/kg, two doses), and the Australian label specifies that in cases of moderate to severe crusted scabies, “more than 3 doses may be required” [5].

### Precedents for interventions that reduce malaria transmission and provide a delayed personal benefit

When used for the treatment of specific diseases such as onchocerciasis, lymphatic filariasis, or in the context of efforts aiming at eliminating these Neglected Tropical Diseases (NTDs), ivermectin provides individuals with a *direct* benefit by reducing their personal parasite burden. It also lowers transmission of NTDs by reducing the parasite burden at community level; this can be seen as an additional *indirect* benefit for the individuals.

Up till recently, the discussion of ivermectin as a tool for malaria had focused exclusively on the potential for indirect benefit. However, there is now limited mouse model data on the potential of ivermectin to directly affect the liver stages of the *Plasmodium* parasite [6, 7], yet the mechanism of action is poorly understood. There has not been specific evaluation of the potential for a direct effect of ivermectin on human *Plasmodium*, although some field data suggest this might be the case [8]. Should it prove to have a direct effect on the malaria parasite, additional regulatory discussions would be needed and possibly be lengthy. These early data, however, suggest that the effect is partial and thus would be a non-primary endpoint, for which label indication would not be sought.

Therefore, for the purpose of this paper, it is assumed that ivermectin mass drug administration (MDA) would reduce malaria transmission at a community level, but healthy individuals would not receive a personal direct benefit from the drug, with the exception being those with susceptible NTDs. There are at least two precedents for such an intervention:

#### Low dose primaquine as gametocytocide

The use of low dose primaquine, together with a course of artemisinin-based combination therapy (ACT), has been recommended by the WHO to reduce transmission in low-transmission areas via its gametocytocidal effect on *Plasmodium falciparum* [9], even in the absence of prophylactic effect or activity against asexual parasites. The recommendation is based on its safety at the recommended dose and on the expected population benefit obtained by the transmission-blocking effect, particularly in areas threatened by artemisinin resistance.

#### Transmission-blocking vaccines. The work of PATH Malaria Vaccine Initiative (MVI)

In 2010, PATH MVI hosted a workshop to explore the possibility of including transmission-blocking vaccines in strategies towards malaria elimination; it focused on the clinical development and regulatory pathways for such a tool. The outcome of that workshop and the progress achieved since 2010 have been updated recently [10]. From the regulatory perspective, there are many parallels between the use of a transmission-blocking vaccine and the use of ivermectin for vector control. Here are some important conclusions that could be applicable to endectocide use in humans [10]:

- Transmission-blocking vaccines are seen as potential tools for accelerating to elimination and possibly, prevent re-introduction.
- The US Food and Drug Administration has indicated that there is no legal bar to prevent the development of transmission-blocking interventions, but ethical review would be critical; that the proposed endpoint of a delayed personal benefit via a community effect is not a major obstacle for clinical development; and that there are two potential clinical development pathways for transmission-blocking vaccines: a cluster randomized trial proving clinical benefits at community level, or approval based on biological surrogates of efficacy and confirmation of efficacy at community level post-approval.
- There is need to standardize the assays and efficacy correlates for transmission-blocking vaccines.
- The delayed personal benefit of transmission–blocking interventions should be the primary emphasis, rather than referring to these as “altruistic”.
- Modelling may help define target efficacy early in the development process and provide insight on the public health benefit in different settings as the added benefit may not be the same in different scenarios.
- Discussions on the manufacture, procurement and distribution for large/remote populations should be started early.

### Key regulatory pathway points for the novel application of ivermectin

#### What is the most appropriate regulatory agency, given the overlap between pharmaceuticals (drug), vector control, and indirect impact on malaria?

The proposed use of the drug ivermectin to reduce malaria transmission by its mosquito-killing effect implies the mass administration of the drug to humans; hence, regulatory approval should come from the drug section of a human health agency. The FDA Center for Biologics has been conceptually supportive of a transmission-blocking vaccine. Moreover, it has stated that it could rule on products not primarily intended to be marketed in the United States. It would also rule on products that would have a community effect leading to delayed personal benefit, a key obstacle for licensure for transmission-blocking vaccines [10]. There is no available data on the status on these discussions with the EMA, although it does have Article 58, which supports its offering an opinion on a product to be used primarily in endemic areas outside of EMA’s primary geographic remit in collaboration with the WHO and relevant non-EU regulatory authorities. Further discussion with both regulatory agencies by a potential sponsor would be required.

#### Potential regulatory pathway for ivermectin for malaria vector indication

If the goal is application for approval for novel use of the licensed product, then regulatory approval for drug repurposing could be sought via the 505(b)(2) pathway [11]. The 505(b)(2) has the advantage of allowing the use of evidence from studies not conducted by the sponsor, alleviating costs and reducing time to approval. An FDA draft guidance specifically for developing treatment and prophylactic products for malaria was drafted in 2007 [12].

In any case, new tools and/or indications need to be proven effective [13] and safe [14]. For an ivermectin-based vector control tool, the best clinical trial design to demonstrate both safety as well as public health impact on malaria transmission is a pivotal cluster randomized trial with sufficient power to assess both key endpoints. It should demonstrate added value on top of standard vector control tools, which should serve as the referent. As the impact and risk/benefit ratio of ivermectin MDA is expected to vary according to the baseline transmission, the selection of the scenario for the first study is key [15]. Note that this design was successfully utilized to definitely demonstrate the impact of other vector control tools, specifically LLINs [16].

For novel applications or new formulations, the FDA expedited approval process could be an option [17]. The FDA Expedited Approval Process aims to “facilitate and expedite development and review of new drugs to address unmet medical need in the treatment of a serious or life threatening condition (using) fast track designation, breakthrough therapy designation, accelerated approval, and priority review designation” [17]. For malaria elimination, both the challenges of residual transmission and insecticide resistance could meet the criterion on unmet medical need, and make ivermectin a good candidate for this approach. Further discussions with regulatory agencies will be needed.

However, the accelerated approval scheme is based on the use of surrogate biological markers of efficacy [18]. In the case of ivermectin, the reduced survival seen in vectors feeding on treated subjects may be an appropriate surrogate marker but is unlikely to lead to regulatory approval, much less policy recommendation and implementation at country level.

### Key efficacy points for licensure

- Key efficacy knowledge gaps were defined in the first paper of this thematic series [19].
- The efficacy of any ivermectin-based regimen and indication is likely to depend on the baseline transmission intensity due to the nonlinear relationship between transmission and clinical malaria [20].
- The efficacy will be a factor of the lethality and duration of effect (both directly related to the dose and formulation used) [19].
- A WHO statement would help define the target efficacy considered to be of public health value. This would be followed by consensus and feedback from each regulatory agency. Importantly, the proportional importance of residual transmission in the pre-elimination setting and the potential contribution of ivermectin as a tool should be considered.

#### Modelling to inform potential efficacy trial design and key parameters

Modelling will play a key role in the pre-licensure stage, when it can provide insight into the needed efficacy threshold to achieve certain goals in different transmission settings, i.e. interrupt transmission and suppress transmission by a target proportion. Additional factors that can be addressed by modelling include target population coverage, target blood levels, and their duration [21–23]. The risk–benefit assessment will vary according to the transmission scenarios.

#### Key efficacy question for regulatory purposes

In elimination campaigns through a high level of community MDA and existing vector control tools, does ivermectin add benefit, i.e. is it a valuable complementary vector strategy? If so by which mechanism? i.e. mosquito killing, partial prophylactic effect, others?

Ivermectin would reduce transmission by suppressing the vector population. It is envisaged as a complementary vector control strategy. Its “transmission-blocking” effect should not be compared with drugs that primarily reduce transmission from human-to-mosquito such as primaquine [15] as the impact of ivermectin is likely to be much higher. The primary outcome of studies assessing transmission-blocking drugs is normally the infectivity of humans to mosquitos as read by the presence of oocysts or sporozoites in mosquitoes fed on treated volunteers [24] while the primary outcome of studies assessing ivermectin and other endectocides in the insectary is usually mosquito survival. These are different but complementary strategies. The challenge for the malaria community is generating the data that enables selection of the most cost-effective strategy for varying strata of malaria endemicity.

#### Key safety points for licensure

Key knowledge gaps were defined in the first paper of this thematic series [19].

Ivermectin has been proven safe in MDA campaigns in the last 30 years, primarily in single dose campaigns distributed once or twice a year. However, its use in malaria is likely to include higher or more frequent doses which may affect the safety profile. There is some guidance on the cut-off points for severe adverse event for anti-malarials intended to be used in MDA campaigns [25].

### WHO prequalification

The WHO prequalification (WHO-PQ) process assures quality, safety, efficacy and suitability of priority medicines for low and middle income countries [26]. The WHO-PQ scheme includes ivermectin among the drugs that can be prequalified for NTDs [27], yet to date no sponsor has submitted their ivermectin product for prequalification. This is likely because the drug used in Onchocerciasis and LF programmes country is donated by Merck [28], i.e. without financial support from funds like the Global Fund to Fight AIDS, Tuberculosis and Malaria (GFATM), hence the product does not require prequalification for its current MDA use. The market price in the Europe is 18.44 euros for four tablets of 3 mg [29], in the US the National Drug Acquisition Cost for Stromectol^®^ in January 2017 was 4.47 US dollars per each 3 mg tablet [30]. No price has been negotiated to the volume requirements for public sector procurement for malaria or NTD MDAs.

## Policy pathway

The uptake of any ivermectin-based strategy by countries will depend on the presence of a clear WHO policy recommendation that is in turn supported by relevant evidence regarding efficacy and safety, as well as data on cost effectiveness, ethics, and community acceptance.

### Role within WHO to assess the use of ivermectin for malaria

Once consensus on settings, comparators and outcome measures of new trials has been reached, evidence would likely be evaluated by the Malaria Policy Advisory Committee (MPAC) of the Global Malaria Programme at WHO. Given the geographic and disease overlaps, the interface between the malaria and NTD programmes will play an important role, and there are precedents for cross-WHO coordination to guide and evaluate product development and policy recommendations.

### Refining the evidence needed for a WHO policy recommendation

It will be important to align the development of any ivermectin-based tool with the unique requirements of health systems of the endemic countries in which it would be used [31]. The type of evidence required during the WHO policy development process has been reviewed by Milstien et al. based on the introduction of malaria intermittent preventive treatment in infancy (IPTi) and four relatively recent vaccines as a case study for new malaria vaccines [32]. Their conclusions were used as guidance for the present section. The evidence needed for a policy recommendation can be classified in four main categories: efficacy, safety, feasibility and cost-effectiveness. Given the particular nature of an ivermectin-based tool to reduce malaria transmission, the category acceptability is also included here.

### Key policy questions

Recommendation of ivermectin will be based on proven efficacy, safety, cost-effectiveness and feasibility for the geographies and populations where it would be used. Pivotal questions related to these four aspects are posed and answered below.

#### Efficacy

##### (i) Is there evidence of an acceptable level of reduction of morbidity and/or mortality in the target populations?

Using transmission-blocking vaccines as a proxy, “there is currently no clinical trial data available to determine the efficacy threshold that would be required to have a clinically beneficial impact on transmission and achieve elimination” [20]. What is considered an “acceptable” efficacy threshold for ivermectin must be estimated with the help of modelling and validated with empirical data during clinical trials? At a minimum, this must be statistically different than the referent (standard vector control and case management) in a well-designed, sufficiently-powered trial, but it should also be of public health relevance. Of note, given the mandate of providing population at risk with either LLINs or IRS any ivermectin MDA trial would be assessing the superiority of the combination which will require larger trial size. This incremental impact will be considered differently depending on the settings. Various epidemiological settings should be tested with priority given to pre-elimination settings where additional new tools are needed to cover the last mile to elimination.

##### (ii) Is the efficacy demonstrated in different malaria endemicity levels?

Different scenarios for the use of ivermectin to reduce malaria transmission have been considered [15], reflecting the variety of malaria endemicity conditions and elimination scenarios in which it will be used. It is possible that the dosage/dosing regimen combinations will need to be optimized to different scenarios. All scenarios cannot possibly be tested prior to recommendation, but a relevant strategy (dose and regimen) could be based on current approaches to MDA (3-day regimes) or, perhaps, an expansion of seasonal dosing schemes such as seasonal malaria chemoprophylaxis (SMC), although this last approach would require adaptation to include all ages rather than just children and drug–drug interaction studies with SMC drugs. An initial approach to the upper limit of ivermectin dose could be based on the cumulative dose recommended to patients with severe crusted scabies (up to seven 200 mcg/kg doses in a month) [33].

##### (iii) Should the use of an endectocide other than ivermectin be considered?

Other existing endectocides tested as mosquitocidal drugs include eprinomectin, selamectin, moxidectin (all available as systemic insecticides for lifestock) [34], spinosad and nitenpyram (available as systemic insecticides for companion animals) [34] and fipronil (available as a spot-on for companion animals but used systemically under experimental conditions) [35].

Some of the advantages of these alternatives include:

- Primarily the possibility of selecting preclinical candidates with considerably longer half-life.
- Possibly reducing concerns about increasing selective pressure on onchocerciasis and soil-transmitted helminths by using the ones with different mode of action.
- Some of the tested endectocides are effective against *Aedes* mosquitoes, which makes them attractive for the control of arboviruses. Ivermectin is not effective against *Aedes* mosquitoes at physiologically relevant concentrations.

Some disadvantages include:

- Most alternatives are early in development, and thus their safety profile in humans would need to be established. Development of any of these drugs would require extensive toxicological and clinical testing both for safety and efficacy. This would be a longer and costly development pathway that could be pursued in parallel with ivermectin.
- Unknown efficacy of new compounds on NTDs.

#### Safety

##### (i) Is the safety profile acceptable?

In the absence of Loa loa co-endemicity, MDA programmes for onchocerciasis control report no severe adverse reactions to ivermectin and their rate of moderate adverse reactions is ≤1.3% [36]. These include ocular irritation, pruritus, rash, pain (general, lymph nodes, headache and joints), dizziness, weakness, fever, ocular irritation, nausea and diarrhoea [36]. In individuals with a high Loa burden (above 30,000 mf/ml) there is risk of severe adverse event including fata encephalopathy. Such high worm loads are more normally associated with areas of high prevalence which are normally avoided by ivermectin MDA campaigns [37]. However novel screening tools may allow a precise exclusion based on individual risk [38].

##### (ii) Is there significant adverse impact on other malaria prevention and treatment strategies?

This could occur through interaction of ivermectin and anti-malarials and should be addressed during development, particularly with ACT and HIV/TB drugs by means of pharmacokinetic studies [19].

##### (iii) What is the safety profile in immunologically compromised groups, i.e. HIV-infected?

Ivermectin can be used to treat crusted scabies and strongyloidiasis in HIV-positive patients. During MDA, individuals are not stratified according to their serological status; only pregnant women, lactating women in the first week after birth, children <90 cm in height (approximately 15 kg) and the severely ill are systematically excluded [39]. The safety questions in high risk groups will be related to the new dose and dosing schemes proposed that are the same as the rest of the population.

#### Acceptability

##### (i) Would an “only” transmission-blocking intervention be acceptable?

The reduction in malaria transmission achieved through ivermectin would mostly derive from mosquito mortality [22], hence ivermectin should be seen as a new paradigm of vector control, as opposed to a transmission-blocking drug that would treat malaria and also decrease transmission [15]. Moreover, as currently envisioned, ivermectin is not a stand-alone tool, but rather a complementary vector control strategy to be added to the emerging elimination strategy. Finally, the use of ivermectin will provide personal benefit in terms of NTDs and ectoparasites. The caveat is animal studies that indicate a direct of effect of ivermectin on Plasmodium liver stages [6, 7]. This is preliminary, intriguing and needs to be better understood, in terms of mechanism and possible effect in humans.

##### (ii) Potential consequences of malaria ivermectin MDA for NTD programmes

**Fig. 1:**
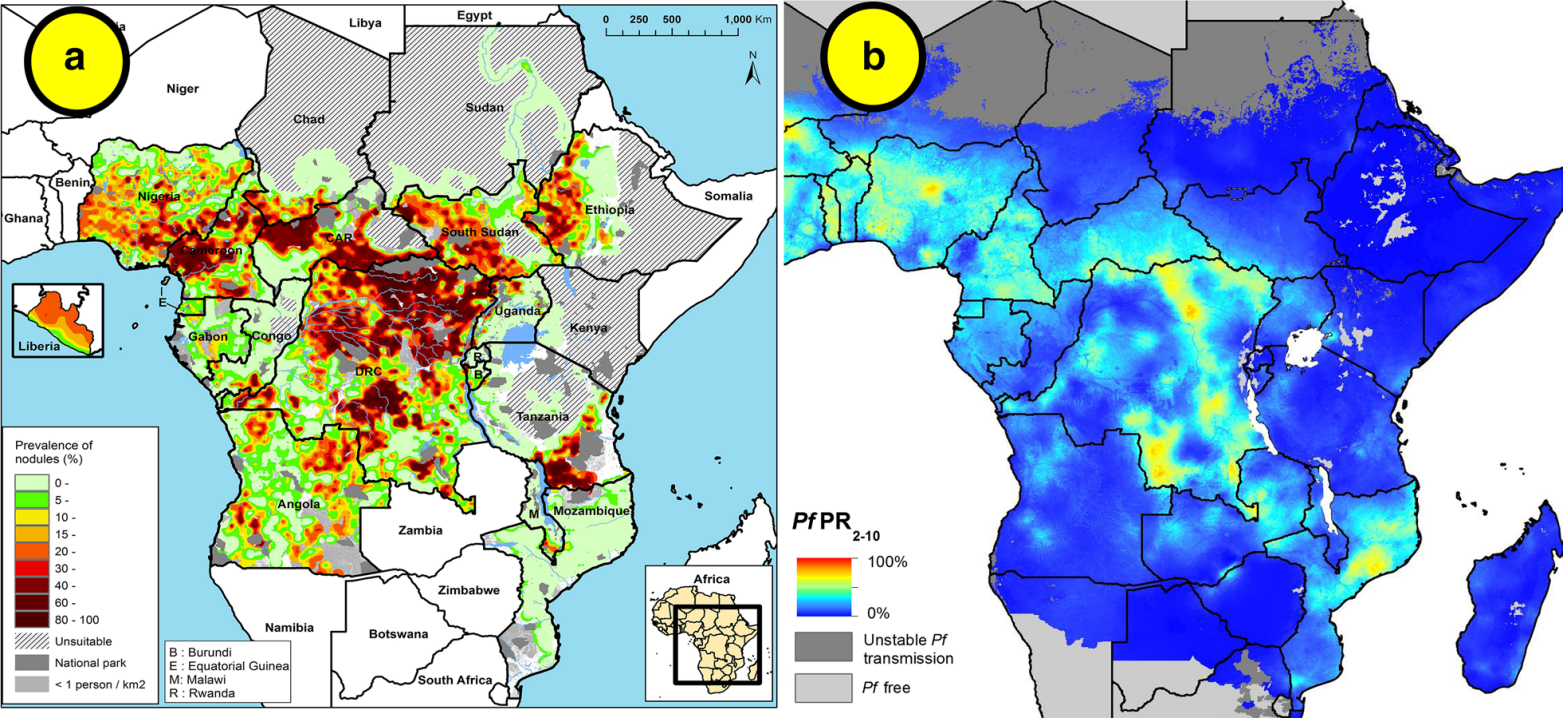
Overlap between selected onchocerciasis and malaria endemic areas in Africa. **a** Estimated prevalence of palpable *Onchocerca* nodules in the 20 African Programme for Onchocerciasis Control countries in 2011 as described by Zouré and colleagues [41]. **b** *Plasmodium falciparum* parasite rate in 2–10 years old in 2015 as described by Bhatt and colleagues [42]

Ivermectin is the drug of choice for the treatment of onchocerciasis. It is also the only drug used in campaigns aimed at eliminating onchocerciasis. In Africa alone, the overlap between onchocerciasis [40] and malaria endemicity [41] is practically 100% as shown in Fig. 1. An increase frequency in the administration of ivermectin (as could be expected if used for malaria) could shorten the time to interrupt transmission of onchocerciasis in certain settings [42] and has been previously advocated as a necessary measure in areas where interruption of transmission has not been achieved after 10 years of annual treatment [43]. If there is potential to shorten the time during which ivermectin donation is needed, this could have profound implications for the business model used today. Moreover, ivermectin has also been demonstrated, in a triple combination, to have remarkable potential impact on elimination lymphatic filariasis [44].

While the single dose used for each of these diseases is not sufficient for impact on malaria, distribution for malaria indication should suffice as a dose for either disease, so careful coordination between malaria and NTD communities would result in most efficient use of supply. Additionally, ivermectin has at least partial activity against several soil-transmitted helminths and ectoparasites, it is reasonable to expect benefit in this context in communities where an ivermectin-based tool for malaria is implemented [45].

This potential tool will optimally require collaboration between the malaria and NTD programmes, including joint research efforts. Two examples or effective collaboration could be:

- Data sharing at programme level to optimize timing of ivermectin distribution for malaria and increase impact (dry vs rainy season) and avoid unnecessary duplication of NTD programmes.
- Ivermectin distribution for malaria with the co-administrations [46, 47] needed as an NTD intervention.

There have been concerns about increasing selective pressure on soil-transmitted helminths and filariae with a wider use of ivermectin. There is limited data on this possibility. Previous reports of ivermectin-resistant *Onchocerca* [48] have been the subject of debate [49–52]. The drug has in fact been used for decades with excellent results in reducing NTD transmission. Additionally, if used in malaria elimination efforts the number of MDA rounds will be limited. There is previous positive experience on the impact of malaria interventions on NTD transmission such as the possibility to halt LF transmission by scaling up LLINs in Nigeria [53].

There is increasing interest in the potential use of Moxidectin for onchocerciasis [54], having a second drug available for onchocerciasis might help manage resistance concerns. However, given the similarities in molecular structure and mode of action [55] there is potential for co-resistance [56]. The lethal concentration 50 of moxidectin for *Anopheles* mosquitoes [34] is one order of magnitude above the Cmax reached using maximum moxidectin doses in humans [57]. In the meantime, ivermectin remains the sole drug for the control and elimination of onchocerciasis and an important pillar for the treatment of lymphatic filariasis.

An additional potential risk is diverting the drug supply away from NTD programmes. Yet this is also an opportunity. A novel indication for malaria would increase market and demand, which should serve as incentive for manufacturers to go through the WHO-PQ process.

##### (iv) What are the expected compliance and adherence? And how could they influence effectiveness?

Effectiveness will be directly related to coverage. Coverage in turn can be greatly influenced by compliance and adherence. Complex and prolonged dosing schemes can negatively impact both [58, 59]. This aspect should be evaluated early through appropriate acceptability studies and addressed by identifying the shortest regimen necessary to have significant impact on malaria transmission.

#### Use of resources

Thus far, more than 2.7 billion doses of ivermectin have been donated and used by involved countries in Africa, Asia and Latin America, administered to more than 80 million people annually and with no cost for commodities to the public sector. The business model of the Mectizan Donation Programme was expanded in 2010 with the commitment of several pharmaceutical companies, along with NGOs, government agencies and academia to sustain, extend and expand the programmes to ensure the necessary supply of donated drugs to help control and eliminate NTDs [60]. The implication of this business model for ivermectin supply for malaria remains to be worked out as there is no commitment for donating the drug for this purpose. New manufacturers are needed to ensure supply for malaria and NTD elimination, and the public sector will need to understand the cost of goods at scale, to best negotiate of supply and price for malaria programmes.

The WHO guidelines for cost-effectiveness analysis of vector control were issued in 1993 and are now archived [61]. Four basic questions are proposed here, the comments on each question reflect the available data at this time.

##### (i) What are the expected costs of protection per person?

The median financial costs of protecting one person for one year with core vector control interventions have been estimated in US$ 2.20 (0.88–9.54) for insecticide treated nets and US$ 6.70 (2.22–12.85) for indoor residual spraying [62]. The most important factor affecting cost of goods for drugs is the clinically effective dose in patients [25]. Ivermectin has the advantage of being effective at low doses (µg/kg), which can reduce costs in comparison with drugs needing doses in the grams range. In the context of the Mectizan Donation Programme, the value for donation of one tablet of ivermectin has been calculated at US$ 1.50 [63]. The purchase price in context of large scale purchase for public sector purchase for malaria MDA will likely be much lower. Ivermectin is off-patent since 1996 and apart from Merck, is available from several generic manufacturers [64], although none of these are yet prequalified by WHO.

The programmatic costs of MDA for onchocerciasis and lymphatic filariasis vary according to geography as well as the method chosen for distribution (passive, community-based, community-directed, national mobile teams) [65].

The fact that the efficacy of ivermectin is directly related to blood concentrations and their duration, its small dose per body weight and its lipophilic nature makes it a good candidate for single-dose, slow-release formulations that can be used to achieve longer term benefits and further reduce costs [23, 25], once development is completed. Once consensus is reached on candidate doses and formulations, packaging discussions should start early as they can greatly influence compliance, costs and programmatic suitability [66].

##### (ii) What financing discussions are needed?

There are important data gaps on what the cost of goods of ivermectin at scale for malaria would be. The upper boundary should be the calculated U.S. donation value for NTDs of US$ 1.5 per 3 mg tablet 1.50 [63], the real price however should be negotiated. The economic benefits of ivermectin distribution for onchocerciasis are partly based on a donated drug. The value of this donated drug may surpass the operational budgets of the control programmes and the economic benefits expected from them for the next 20 years [64]. Given the higher burden and economic costs related to malaria and expected price negotiations, this balance might be more positive, especially in the context of elimination.

An important economic discussion would be the possibility for any new ivermectin-based tool to be financed by the GFATM in case it is included in a country plan, and recommended by WHO. WHO prequalification of the new indication for malaria or any new formulation will be a prerequisite for policy recommendation and thus GFATM financing.

#### Supply

##### (i) Is the manufacture process scalable?

Ivermectin is semi-synthetic derivate of a bacterial bio product [67]. The manufacturing process is technically scalable. As the global demand increased, its production has been enhanced and purified by a number of methods [68, 69]. The current global production is above 150 tons of active pharmaceutical ingredient per year (estimate from the Argentinian Chamber of Veterinary Products, pers. comm.), most of it is for veterinary use. As guidance, only 2.24 tons per year are needed to treat 80 million people, the target of the Mectizan Donation Programme (assuming an average weight of 70 kg, at the 200 µg/kg-dose, twice a year); that is less than 1.5% of the current global production. Even a tenfold increase on the global demand for human use, due to its theoretical application in malaria control would represent less than 15% of the current production due to the co-endemicity of onchocerciasis and malaria in many regions. Note that malaria use would likely be phased in over time.

Here it is important to distinguish between the production of the API (which would be the main limiting step where there an increase in the global demand due to malaria use), and the manufacturer of final products. There are dozens of large scale API producers, mainly in china with some of them reporting an annual production above 50 tons (see Additional file 1). The API used for veterinary and human products can come from the same source but must fulfil different quality standards which might require additional purification steps. Although there are several hundred manufacturers of final product (see for example [70] for a list with more than 100 generic products and manufacturers only in India), the production output and technical capacity of these manufacturers to obtain WHO-PQ will play a key role on ultimately fulfilling the global demand.

##### (ii) Current and prospective global demand for NTDs

More than 200 million doses were donated for the control and eradication of onchocerciasis and lymphatic filariasis in 2015 [28]. The demand will vary according to the operational goals for onchocerciasis (control, elimination or eradication). One estimate is it could reach up to 2.63 billion treatments for the 2013–2045 period [71], but higher demand due to accelerated LF elimination with drug combo strategies can be expected [44, 47].

##### (iii) What would be the Go/No-Go criteria for the development of new formulations or novel dosing schemes?

The efficacy threshold is expected to be in direct relationship with the total dose and the area under the curve [15]. The safety and programmatic feasibility of schemes requiring high or multiple doses should be measured against the expected efficacy. Novel formulations could simplify the dosing schemes and increase compliance but would require R&D investment.

## A note on roles and sequence

This paper looks at the potential use of ivermectin for malaria from a regulatory and policy point perspective. Several key steps needed to fill these gaps have been identified. There are specific actors associated with some activities, for example prequalification can only come from the WHO-PQ team. Other activities have a broader range of potential actors, for example, there are several first tier regulatory authorities qualified to review this approach (EMA, FDA, TGA…). Although some concrete actor-action pairs have been mentioned in the corresponding section of this paper, including redundant lists of potentially involved institutions or committees at each step has been avoided.

There is clear need for a regulatory sponsor to drive this forward on the global health stage. This gap could be filled by a producer interested in the potential novel market, a consortium interested in filling the knowledge gaps or an NGO focused on global health.

**Fig. 2:**
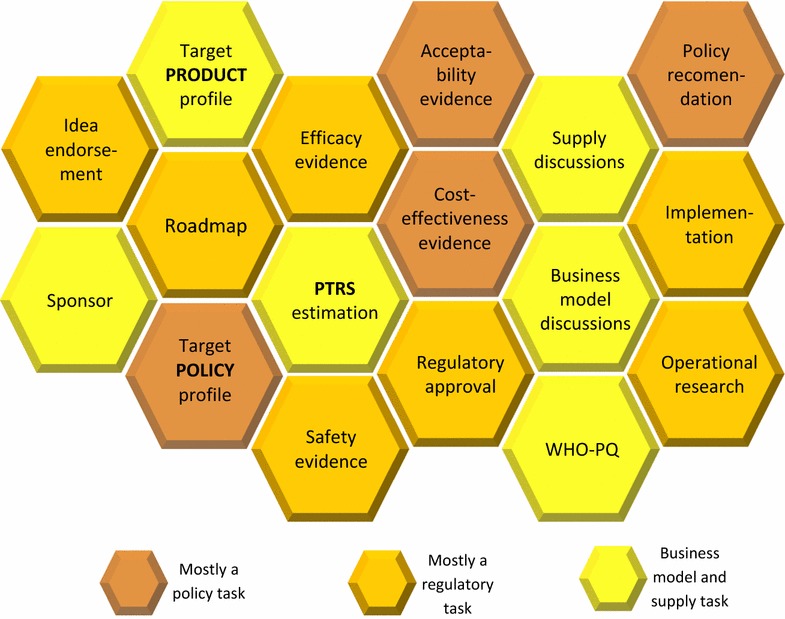
Regulatory, policy and business model tasks related to the development of ivermectin as a complementary tool to reduce malaria transmission. The intended order is *left to right* but the figure should illustrate the fact that some processes will be conducted in parallel and some sequentially. *PTRS* probability of technical and regulatory success, *WHO-PQ* WHO prequalification

The series of concrete actions ultimately leading to implementation have been discussed in two distinct sections, regulatory and policy. This division has been introduced to facilitate the discussion of each particular action, yet the separation is artificial as there are cross-cutting issues related to both pathways. Additionally, it would be a very complex task to try to provide a particular order in which these actions should be followed. It is clear for example that regulatory approval can only follow evidence on efficacy and safety, other points like WHO-PQ for example, would require discussions related to programmatic suitability and technical capacity of producers from early on in the development process. Figure 2 is not intended as a technical guidance for a particular order, but illustrates how several processes are related and could happen in parallel.

## Conclusions

Implementation of an ivermectin-based strategy to reduce malaria transmission will require higher or more frequent doses that currently used for NTDs. Efficacy and safety will be the most important parameters to be evaluated by any stringent regulatory authority; both are directly related to the dose and dosing scheme selected for malaria. For a WHO policy recommendation, additional factors such as cost-effectiveness, acceptability and programmatic suitability will need to be addressed.

## Additional file

**Additional file 1.** Main ivermectin API producers.

## Abbreviations

ACT: artemisinin-based combination therapy
EMA: European Medicines Agency
FDA: US Food and Drug Administration
GFATM: Global Fund to Fight AIDS, Tuberculosis and Malaria
LF: lymphatic filariasis
MDA: mass drug administration
MDP: Mectizan Donation Programme
MPAC: Malaria Policy Advisory Committee
NTDs: neglected tropical diseases
NMCP: National Malaria Control Programme
VCAG: Vector Control Advisory Group
