# Supplementary material for: Ivermectin to reduce malaria transmission III. Considerations regarding regulatory and policy pathways
Source: Malar J. 2017 Apr 24;16:162. doi: 10.1186/s12936-017-1803-2 (PMC5402052; doi:10.1186/s12936-017-1803-2)
Supplement: Supplementary file 1 — Additional file 1. Main ivermectin API producers. [file 12936_2017_1803_MOESM1_ESM.docx]

| **Manufacturers** | **Large scale manufacturers*** | **Manufacturers and traders** |
| --- | --- | --- |
| Ningxia Zoohance Biotech Co., LTD  Nanjing Bangnuo Biotechnology Co., LTD  Zhuhaishi Shuangbojie Technology Co., LTD  Chengdu Newsun Crop Science Co., LTD  Shenzhen Shijingu TechnologyCo., LTD  Hubei Yuancheng Saichuang Technology Co., LTD  Zhuahishu Shuangbojie Technology Co., LTD  Wuhan Hengwo Scien-Tech Co., LTD | Zhejiang Hisun Pharmaceutical Co., LTD  Shandong Qilu King-Phar Pharmaceutical Co., LTD  Hebei Vian Bio-Chemical Co., LTD  Hebei Veyong Animal Pharmaceutical Co., LTD | Chengdu Xinheng Pharmaceutical Co., LTD  Orient Resources International Co., LTD  Shijiazhuang Jiangshan Animal Pharmaceutical Co., LTD  Hebei New Century Pharmaceutical Co., LTD  Shandong Unovet Pharmaceutical Co., LTD  Shijiazhuang Shimu Animal Pramaceutical Co., LTD  Chengdu Qiankun Veterinary Pharmaceutical Co., LTD  Wuhan Benjamin Pharmaceutical Chemical Co., LTD  Xi´an Leader Biochemical Engineering Co., LTD  Changzhou ComWin Fine Chemicals Co., LTD  Hefei Reachever Import and Export Co., LTD  Shaanxi Guanjie Technology Co., LTD  Shijiazhuang ZDHF Stock Raisin Co., LTD  Wuhan Yuancheng Gongchuang Technology Co., LTD  Hanzhou Tianlong Biotechnology Co., LTD  Qingdao Fraken International Trading Co., LTD |

**Additional file 1**. Main ivermectin API producers

*Self-reported production more than 50 tons a year (data provided by each company after direct consultation)
